# Insights into the epidemiological analysis of subarachnoid hemorrhage burden and trends in middle-aged and elderly populations: a global perspective from the Global Burden of Disease Study 2021

**DOI:** 10.3389/fneur.2025.1518319

**Published:** 2025-08-18

**Authors:** Guiming Chen, Yuzhou Cai, Meng Ju, Xiye Zheng, Peng Bai

**Affiliations:** ^1^Interventional Medicine Department, The First Affiliated Hospital of Kunming Medical University, Kunming, Yunnan, China; ^2^Department of Gastrointestinal Surgery, The First Affiliated Hospital of Kunming Medical University, Kunming, Yunnan, China

**Keywords:** subarachnoid hemorrhage, Global Burden of Disease, health workforce, temporal trends, emergency medical services

## Abstract

**Background:**

Subarachnoid hemorrhage (SAH) represents a critical neurological emergency with substantial morbidity and mortality, particularly affecting middle-aged and elderly populations. While previous studies have documented SAH epidemiology, the relationship between health workforce distribution and SAH burden remains largely unexplored globally. This study analyzed global epidemiological patterns, temporal trends, and novel associations between health workforce categories and SAH burden among adults aged ≥ 45 years from 1990 to 2021.

**Methods:**

We utilized Global Burden of Disease (GBD) Study 2021 data across 204 countries and territories. We calculated age-standardized incidence rates (ASIR), age-standardized prevalence rates (ASPR), age-standardized mortality rates (ASMR), and age-standardized disability-adjusted life years (DALYs) rates (ASDR). Estimated annual percentage changes (EAPCs) assessed temporal trends. We performed age-period-cohort analysis, decomposition analysis, examined associations with socio-demographic index (SDI), conducted frontier analysis, and explored correlations between 22 health workforce categories and SAH burden.

**Results:**

Globally, SAH incident cases increased from 321,512 to 487,851 (51.7% increase), while prevalent cases rose from 3,074,793 to 5,654,572 (83.9% increase) between 1990 and 2021. Despite increasing absolute numbers, age-standardized rates declined: ASIR (EAPC: −0.65%), ASPR (EAPC: −0.08%), ASMR (EAPC: −1.39%), and ASDR (EAPC: −1.31%). East Asia showed the highest burden. Middle SDI countries had the highest ASIR and ASMR. Decomposition analysis revealed population growth as the primary driver of case increases (190.28% for incidence), while epidemiological improvements caused substantial reductions (−99.26% for incidence). Our novel health workforce analysis revealed that emergency medical workers demonstrated strengthening protective associations with mortality outcomes over three decades, with correlations improving from r = −0.21 to r = −0.30 (*p* < 0.001) for deaths. Countries with highest SAH mortality had remarkably low emergency medical worker densities (0.47–11.2 per 10,000 population) compared to low-mortality countries (1.7–26.1 per 10,000 population).

**Conclusion:**

Despite increasing absolute SAH burden, age-standardized rates declined globally, indicating epidemiological improvements. Our novel finding of strong inverse relationships between emergency medical workforce density and SAH mortality provides the first global evidence for targeted healthcare capacity building. These findings offer new insights for optimizing healthcare resource allocation and reducing global SAH burden, particularly in regions with inadequate emergency medical infrastructure.

## Introduction

Subarachnoid hemorrhage (SAH) is a rare but highly fatal cerebrovascular disease with a relatively low incidence. However, its sudden onset and high lethality (up to 40%) make it a significant public health challenge on a global scale (1–3). Data from the Global Burden of Disease 2021 indicate that approximately 500,000 individuals worldwide are affected by SAH each year (4). Those who survive often experience long-term neurological deficits that significantly impair their quality of life and daily independence, thereby contributing to the dual social and economic burden (5, 6). The global burden of the disease varies significantly by region, gender and age group, as does the global health system response to the disease (7, 8). Despite recent advances in the early diagnosis and treatment of SAH due to advances in medical technology, particularly in high-income countries, morbidity and mortality remain high in low- and middle-income countries (9).

In light of the global trend of population aging, the middle-aged and elderly population aged 60 years and above has emerged as a high-risk group, exhibiting a persistently increasing burden of subarachnoid hemorrhage (8, 10). The prevalence of chronic diseases, such as hypertension and atherosclerosis, is higher among older adults, and these conditions, in conjunction with increased vascular fragility, markedly elevate the risk of SAH. Furthermore, older women are particularly susceptible to cardiovascular disease due to the impact of hormonal changes, particularly the decline in postmenopausal hormones, which contribute to an increased risk of SAH. The neurological recovery of the elderly is significantly less robust than that of younger patients, resulting in markedly elevated rates of SAH-related disability and mortality (11). Consequently, in the context of an aging global population and an unequal distribution of healthcare resources, comprehensive studies focusing on this specific demographic are of paramount importance.

Despite the recent increase in research on subarachnoid hemorrhage, epidemiological studies on middle-aged and older populations remain limited. Furthermore, these studies have largely focused on the overall population or specific pathological mechanisms (12, 13). In low- and middle-income countries, older populations are more challenging to prevent and treat due to restricted access to healthcare and a dearth of data. Importantly, while healthcare workforce capacity has been recognized as a critical determinant of health outcomes, the relationship between health workforce distribution and SAH burden has remained largely unexplored at the global level, representing a significant knowledge gap in understanding modifiable healthcare system factors that could influence SAH outcomes. To address this research gap, we employed the Global Burden of Disease Study 2021 dataset to conduct comprehensive analyses of the incidence, prevalence, mortality, and disability-adjusted life years associated with subarachnoid hemorrhage in middle-aged and older populations. Additionally, we employed disaggregated and predictive techniques to examine potential future trends, and conducted novel analyses examining associations between health workforce categories and SAH disease burden across countries and over time, with the aim of informing global health policy and making significant contributions to the existing literature.

## Methods

### Data source

The Global Burden of Disease Study 2021 offers a comprehensive assessment of global health losses, employing the latest epidemiological data and standardized methodologies. The study encompasses 371 diseases and injuries and 88 risk factors across 204 countries and territories, with disaggregation by age and gender to facilitate a more comprehensive understanding of global health challenges. In the case of subarachnoid hemorrhage in middle-aged and older populations, the GBD study employed the Bayesian meta-regression tool DisMod-MR 2.1, which integrates multiple data sources with the objective of ensuring the accuracy and reliability of the results. For further details on these methods, please refer to the following publications (14, 15). In this study, we extracted estimates of incidence, prevalence, deaths, and disability-adjusted life years associated with subarachnoid hemorrhage in middle-aged and older adults from the GBD 2021 dataset, with 95% uncertainty intervals. To facilitate meaningful cross-regional comparisons, rates per 100,000 population were standardized. To ensure consistency, Age-Standardized Rates were calculated based on a global population model (16). Furthermore, the findings were analyzed using a sociodemographic index that combines factors such as income, education and fertility levels to classify areas into five classes. This provides an important framework for assessing the impact of sociodemographic development on health outcomes (17).

### Age group definitions

Following established epidemiological conventions and previous Global Burden of Disease studies focusing on cerebrovascular disease in aging populations, we defined “middle-aged and older adults” as individuals aged 45 years and above. This age threshold is consistent with international guidelines recognizing 45 years as the onset of elevated cerebrovascular risk and is widely used in cardiovascular and stroke epidemiology research. The 45-year lower age limit is supported by epidemiological evidence showing that while subarachnoid hemorrhage can occur at any age, the incidence begins to increase notably after age 45, with particular elevation in risk factors such as hypertension, atherosclerosis, and hormonal changes, especially in postmenopausal women.

For age-specific analyses, we utilized 5-year age groups: 45–49, 50–54, 55–59, 60–64, 65–69, 70–74, 75–79, 80–84, 85–89, 90–94, and 95+ years. These standardized age intervals are consistent with Global Burden of Disease methodology and provide sufficient sample sizes for reliable estimates while maintaining adequate granularity to detect meaningful age-related trends across the adult lifespan. This age stratification system aligns with World Health Organization recommendations for cardiovascular disease surveillance in aging populations and ensures our findings are comparable with other major epidemiological studies in this field.

### Descriptive and trends analysis

In order to evaluate the impact of subarachnoid hemorrhage on middle-aged and older adults on a global scale, we conducted a comprehensive analysis of global, regional and country-level data between the years 1990 and 2021. The study encompassed an examination of the number of cases, incidence, prevalence, and Age-Standardized Rates (ASRs) of YLD (years of disability-adjusted life), with a focus on the tracking of change in trends over a period of 32 years. By comparing data from five Sociodemographic Index (SDI) regions, 21 GBD regions, and 204 countries and territories, we were able to identify differences between the regions. The trends were quantified using estimated average percentage change (EAPC), based on a linear regression model (18). Positive values of EAPC indicate an increasing trend in prevalence, while negative values indicate a decreasing prevalence. Each EAPC value is accompanied by a 95% confidence interval (CI). A positive value for the lower limit of the CI indicates an increasing trend, a negative value indicates a decreasing trend, and a value within the 95% CI range indicates stability.

### Frontier analysis and control effects calculation

We conducted frontier analysis to evaluate the control effectiveness of SAH management across countries with varying socio-demographic development levels. The frontier represents the optimal performance (lowest disease burden) achievable at each SDI level, determined by identifying the best-performing countries within sliding SDI windows. For each disease burden measure (incidence, prevalence, mortality, and DALYs), we constructed the frontier using locally weighted scatterplot smoothing (LOESS) regression to establish the minimum achievable rates at each SDI level. Control effectiveness was quantified by calculating the efficiency difference between each country’s actual performance and the frontier benchmark using the formula: Efficiency Difference = (Actual Rate − Frontier Rate)/Frontier Rate × 100. Countries with efficiency differences close to zero demonstrate optimal control effectiveness for their development level, while larger positive values indicate suboptimal performance and potential areas for improvement. This methodology allows for fair comparisons of healthcare system performance by accounting for underlying socio-economic constraints.

The disparity assessment was completed using the slope inequality index (SII) and the concentration index (CI). A positive value of the SII indicates that health is relatively better in areas with a higher SDI, while the CI fluctuates between −1 and 1, reflecting equity in the distribution of health resources. The objective of the decomposition analyses was to identify the underlying factors that have contributed to changes in the incidence, prevalence, mortality, and disability-adjusted life years of stroke in middle-aged and older adults between 1990 and 2021. Particular attention was paid to the influence of population growth and aging.

### Health workforce correlation analysis

To examine the association between health workforce distribution and subarachnoid hemorrhage disease burden, we conducted correlation analyses using data from the Global Health Observatory (GHO) and Global Burden of Disease (GBD) Study 2019. Health workforce data were obtained from the IHME GBD 2019 Human Resources for Health dataset, which provides standardized estimates of health worker densities per 10,000 population across 22 distinct health worker categories for 204 countries and territories in 1990 and 2019. These categories encompass diverse healthcare professionals including physicians, clinical officers, community health workers, nursing and midwifery professionals, emergency medical workers, pharmacists, dentists, physical therapists, and other specialized health personnel (19).

### Statistical analysis

The relationship between the burden of subarachnoid hemorrhage in middle-aged and elderly people and the SDI at five socio-demographic index (SDI) levels was analyzed in 21 GBD districts and 204 countries using a smoothed spline model. Concurrently, we evaluated the linear correlation between the variables through the application of Pearson’s correlation coefficient (20, 21). The SDI ranges from 0 (least developed) to 1 (most developed), combining factors such as per capita income, years of education, and fertility to reflect the impact of socio-economic development on the burden of SAH in middle-aged and older adults. The disparity assessment was completed using the slope inequality index (SII) and the concentration index (CI). A positive value of the SII indicates that health is relatively better in areas with a higher SDI, while the CI fluctuates between −1 and 1, reflecting equity in the distribution of health resources (22–24). The objective of the decomposition analyses was to identify the underlying factors that have contributed to changes in the incidence, prevalence, mortality, and disability-adjusted life years of stroke in middle-aged and older adults between 1990 and 2021. Particular attention was paid to the influence of population growth and aging (25). Furthermore, we employed frontier analyses to ascertain the burden of SAH in each region in comparison to the most optimal performing regions. This enabled the identification of areas for improvement and provided a scientific foundation for the implementation of pertinent policy interventions. For prospective burden projections, we utilized Bayesian age-period-cohort (BAPC) analyses. All analyses presented in this paper were conducted via the R language (version 4.3.2).

## Results

### Global burden of subarachnoid hemorrhage

A comprehensive descriptive analysis of the global incidence, prevalence, mortality, and disability-adjusted life years (DALYs) is presented. The data from 2021 indicate that subarachnoid hemorrhage represents a substantial health burden on a global scale, particularly affecting the middle-aged and elderly population.

Compared to 1990, the estimated number of incident cases increased from 321,512 (95% UI: 225,805 to 449,754) to 487,851 (95% UI: 356,037 to 660,192) in 2021, representing an increase of 51.7%. The number of prevalent cases increased from 3,074,793 (95% UI: 2,624,046 to 3,593,409) to 5,654,572 (95% UI: 4,943,325 to 6,465,181), representing an 83.9% increase. In 2021, approximately 312,860 deaths (95% UI: 265,096 to 363,366) and 8,036,753 DALYs (95% UI: 6,972,612 to 9,272,699) were attributed to subarachnoid hemorrhage in the middle-aged and older populations worldwide.

In 2021, the age-standardized incidence rate (ASIR), age-standardized prevalence rate (ASPR), age-standardized mortality rate (ASMR), and age-standardized DALYs rate (ASDR) for subarachnoid hemorrhage in middle-aged and older adults per 100,000 population were 20.68 (95% UI: 15.13–27.93), 236.03 (95% UI: 206.17–270.01), 13.47 (95% UI: 11.40–15.64), and 336.93 (95% UI: 292.07–388.66), respectively (Supplementary Table S15).

### Burden by socio-demographic index, region, and country

At all socio-demographic index (SDI) levels, the number of cases of subarachnoid hemorrhage in middle-aged and elderly populations showed year-by-year increases. The incidence in high SDI areas (106,753 cases) and medium-high SDI areas (170,752 cases) was significantly higher than that in low SDI areas (26,501 cases), with similar distribution patterns observed across SDI quintiles.

The subarachnoid hemorrhage-related mortality and DALYs in the middle-aged and elderly population showed an upward trend in high, medium-low, and low SDI areas, while a downward trend was observed in medium and medium-high SDI areas. Notably, the number of deaths (119,369 cases) and DALYs (3,011,173 years) in medium SDI areas were relatively high.

**Figure 1:**
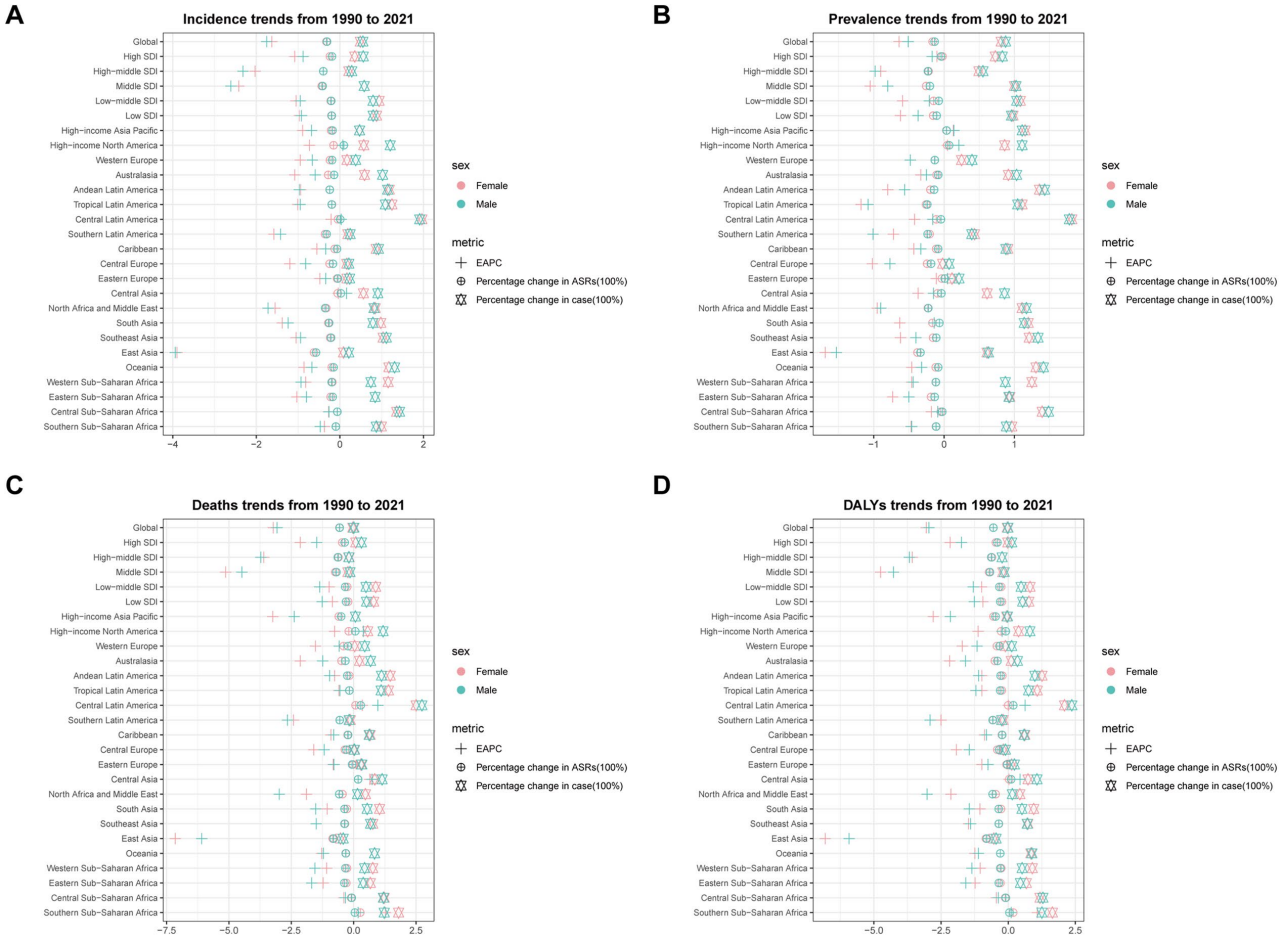
The estimated annual percentage change (EAPC) in age-standardized incidence rate (ASIR), age-standardized prevalence rate (ASPR), age-standardized mortality rate (ASMR), and age-standardized disability-adjusted life years (DALYs) rate (ASDR) of subarachnoid hemorrhage among middle-aged and elderly populations by sex across socio-demographic index (SDI) quintiles and Global Burden of Disease (GBD) regions from 1990 to 2021. **(A)** Age-standardized incidence rate; **(B)** Age-standardized prevalence rate; **(C)** Age-standardized mortality rate; **(D)** Age-standardized DALYs rate.

From 1990 to 2021, the age-standardized rates of subarachnoid hemorrhage incidence, prevalence, mortality, and DALYs showed decreasing trends across all SDI levels. In 2021, the moderate SDI region had the highest ASIR at 23.28 (95% UI: 16.93 to 31.41) and ASMR at 17.23 (95% UI: 13.50 to 20.66), with their estimated annual percentage changes (EAPCs) being −2.22 (95% CI: −2.47 to −1.97) and −4.69 (95% CI: −5.15 to −4.23), respectively. The highest ASPR was observed in high SDI regions at 306.88 (95% CI: 273.63 to 344.35), with an EAPC of −0.20 (95% CI: −0.25 to −0.15). The ASDR was highest in the low-to-medium SDI region at 412.40 (95% UI: 318.95 to 537.93), with an EAPC of −1.31 (95% CI: −1.34 to −1.27) (Supplementary Table S15; Figure 1).

At the Global Burden of Disease (GBD) regional level, there was an overall increase in incidence and prevalence across all regions. Mortality and DALYs decreased only in specific regions, such as Central Europe and Latin America. In 2021, the burden of subarachnoid hemorrhage was highest in the East Asia region, with 123,620 incident cases (95% UI: 87,494 to 169,931), 1,055,188 prevalent cases (95% UI: 871,036 to 1,270,303), 90,564 deaths (95% UI: 64,225 to 116,338), and 2,077,302 DALYs (95% UI: 1,532,659 to 2,617,422). This was followed by South and Southeast Asia (Supplementary Table S15; Supplementary Figure 1).

Notably, East Asia also demonstrated the largest decreases in age-standardized rates for all burden measures. In 2021, the EAPCs for ASIR, ASPR, ASMR, and ASDR in this region were −3.60 (95% CI: −3.97 to −3.22), −1.59 (95% CI: −1.70 to −1.48), −6.56 (95% CI: −7.21 to −5.91), and −6.17 (95% CI: −6.76 to −5.57), respectively (Supplementary Table S15; Figure 1; Supplementary Figure 2).

At the country level, the three countries with the highest incidence of subarachnoid hemorrhage in middle-aged and older adults in 2021 were China [118,466 cases (95% UI: 83,589 to 163,257)], India [62,206 cases (95% UI: 43,216 to 87,117)], and Japan [32,982 cases (95% UI: 24,119 to 44,199)]. The Solomon Islands [95% UI: 60.20 (44.91 to 79.19)], Micronesia [45.87 (95% UI: 34.40 to 60.25)], and Kiribati [45.34 (95% UI: 33.85 to 59.61)] had the highest ASIR globally. China [87,461 (95% UI: 61,119 to 113,547)] and India [41,178 (95% UI: 28,358 to 58,728)] had the highest total deaths, while the highest ASMR were found in Mongolia [42.05 (95% UI: 28.26 to 58.65)], Honduras [38.46 (95% UI: 23.83 to 57.23)], and Solomon Islands [37.34 (95% UI: 20.99 to 61.59)]. For DALYs, China [1,995,371 years (95% UI: 1,449,841 to 2,535,999)] and India [1,210,158 years (95% UI: 869,511 to 1,672,642)] had the highest burden. The countries with the highest ASDR were Haiti [973.21 (95% UI: 449.43 to 1,715.21)], Solomon Islands [965.86 (95% UI: 583.29 to 1,530.68)], and Honduras [956.45 (95% UI: 605.78 to 1,406.53)] (Supplementary Table S1; Supplementary Figure 3).

**Figure 2:**
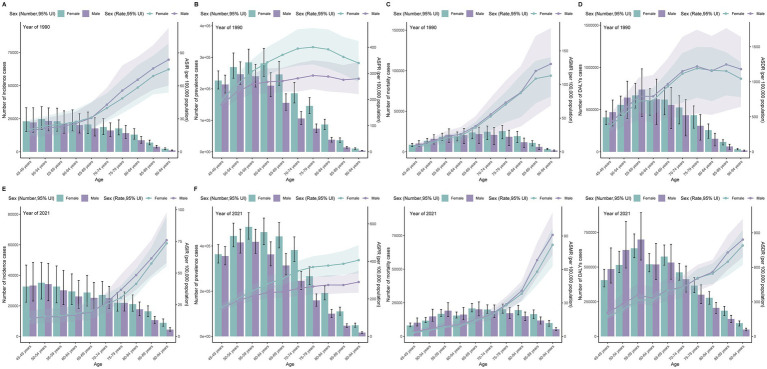
Estimated annual percentage change (EAPC) for age-standardized incidence rate (ASIR), age-standardized prevalence rate (ASPR), age-standardized mortality rate (ASMR), and age-standardized DALYs rate (ASDR) of subarachnoid hemorrhage among elderly men and women across 204 countries and territories from 1990 to 2021. **(A)** EAPC of ASIR; **(B)** EAPC of ASPR; **(C)** EAPC of ASMR; **(D)** EAPC of ASDR.

From 1990 to 2021, the incidence, prevalence, mortality, and DALYs of subarachnoid hemorrhage showed decreasing trends in most countries. The largest decreases in incidence were observed in China and Iraq, with EAPCs of −4.01% (95% CI: −4.42 to −3.60) and −2.54% (95% CI: −2.77 to −2.30), respectively. For prevalence, the largest decreases were in China and Ireland, with EAPCs of −4.01% (95% CI: −4.42 to −3.60) and −2.02% (95% CI: −2.15 to −1.89), respectively. The largest reductions in mortality were observed in China and Lebanon, with EAPCs of −4.01% (95% CI: −4.42 to −3.60) and −4.91% (95% CI: −5.20 to −4.62), respectively. Similarly, the reductions in DALYs were most significant in China and Lebanon, with EAPCs of −4.01% (95% CI: −4.42 to −3.60) and −4.83% (95% CI: −5.14 to −4.51), respectively (Supplementary Table S1; Figure 2).

### Age- and sex-specific burden patterns

Data from 2021 demonstrate that globally, the number of subarachnoid hemorrhage cases in middle-aged and older people is slightly higher in women than in men. The burden shows significant differences across age groups, with incidence and mortality rates peaking in the 50–54 and 65–69 age groups, while prevalence and DALYs are highest in the 55–59 age group.

**Figure 3:**
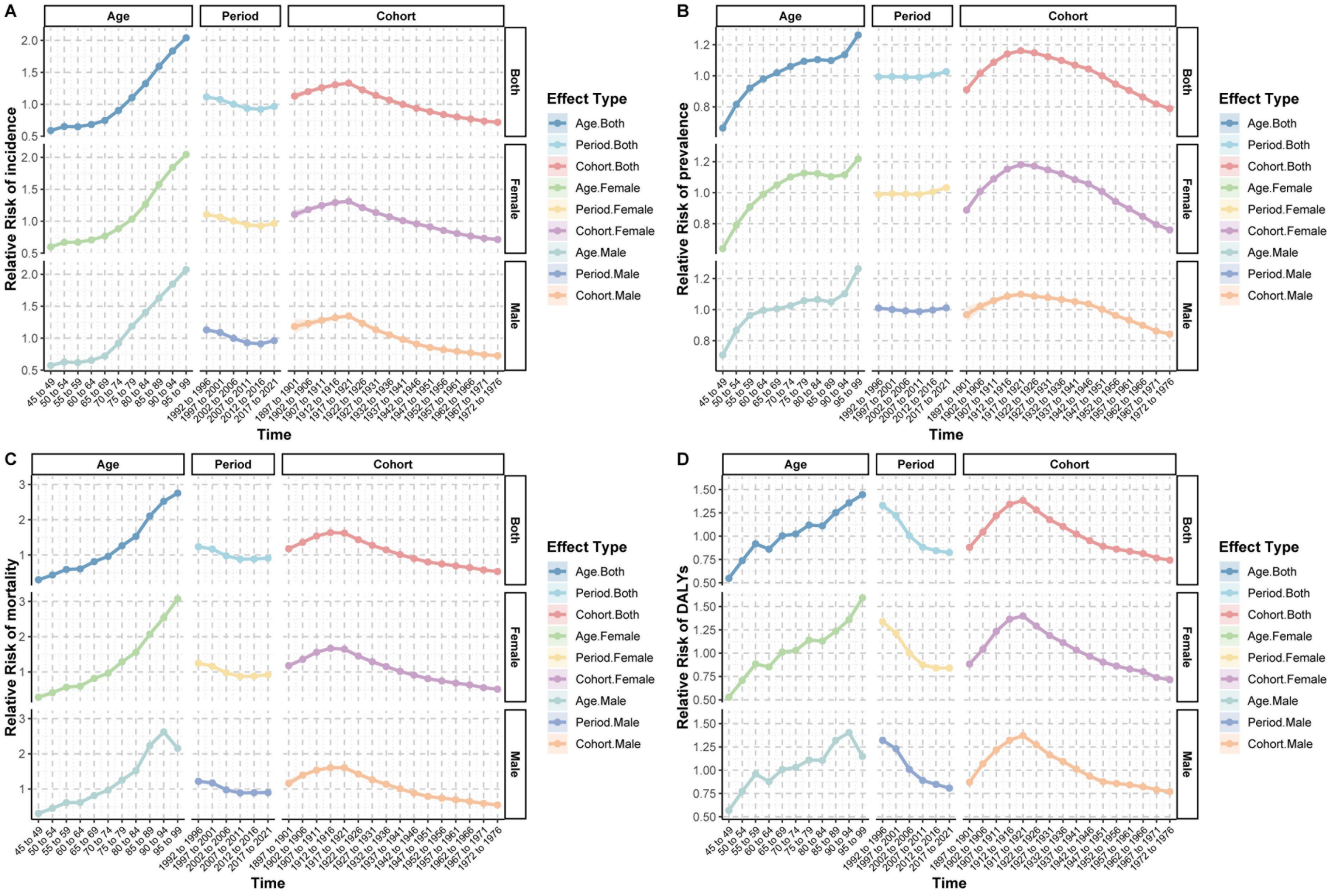
Age-specific patterns of subarachnoid hemorrhage burden among middle-aged and elderly populations by 5-year age groups (45–49 to 90–94 years) in 1990 and 2021. **(A,E)** Incidence cases and age-standardized incidence rate; **(B,F)** Prevalence cases and age-standardized prevalence rate; **(C,G)** Deaths and age-standardized mortality rate; **(D,H)** Disability-adjusted life years (DALYs) and age-standardized DALYs rate.

In patients aged 50 years and older, the incidence and prevalence were higher in women than in men. Although mortality and DALYs were slightly higher in men than in women in the 50–65 age group, they were significantly higher in women than in men in the 65 + age group. Additionally, the age-standardized rates of incidence, prevalence, mortality, and DALYs gradually increased with age in the middle-aged and elderly groups across all SDI regions. The overall burden was concentrated in the 50–69 age group. Compared with 1990, all age-standardized rates showed decreasing trends in 2021 (Supplementary Table S2; Figure 3).

### Age-period-cohort analysis

**Figure 4:**
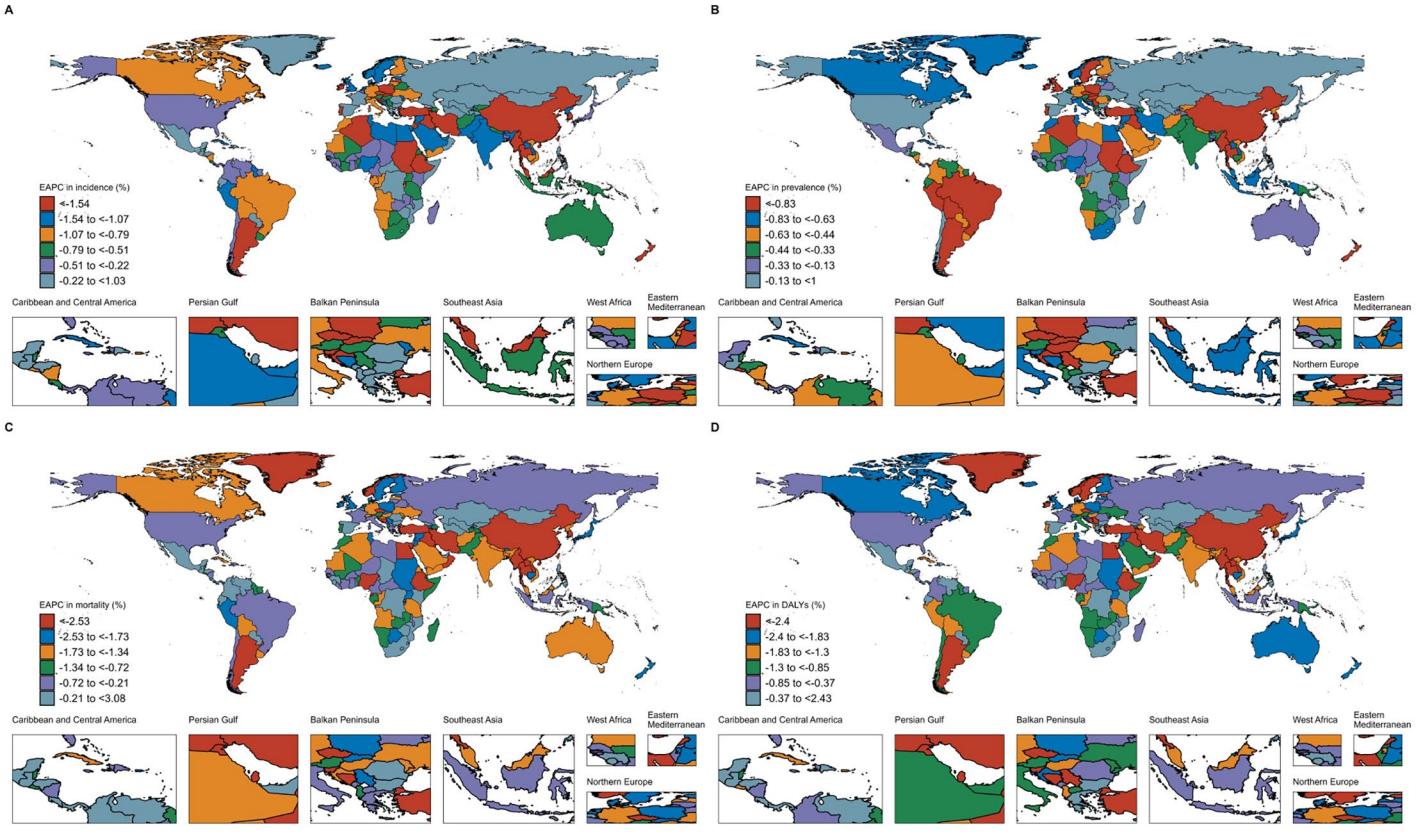
Age-period-cohort analysis of relative risk (RR) for incidence, prevalence, mortality, and disability-adjusted life years (DALYs) of subarachnoid hemorrhage among middle-aged and elderly populations. **(A)** Incidence relative risk; **(B)** Prevalence relative risk; **(C)** Mortality relative risk; **(D)** DALYs relative risk.

The age-period-cohort analysis revealed distinct temporal patterns for subarachnoid hemorrhage burden. After adjusting for period and cohort effects, the age effect showed a significant upward trend in incidence rate with increasing age, particularly after 65–69 years, which increased sharply. After controlling for cohort and age effects, the period effect on incidence rate showed a slight decrease from 1992 to 1996 (RR = 1.16) to 2017–2021 (RR = 0.97). The cohort effect demonstrated that disease risk gradually increased from 1897 to1901 (RR = 1.13) to a peak in 1912–1916 (RR = 1.33) and then gradually decreased (Supplementary Table S3; Figure 4A). Similar trends were observed for ASMR and ASDR (Figures 4C,D).

The effect of age on prevalence showed a gradual increase in relative risk from 45 to 49 years (RR = 0.66) to 95–99 years (RR = 1.26). After adjustment for cohort and age effects, the period effect on prevalence increased from 1992 to 1996 (RR = 0.99) to 2017–2021 (RR = 1.03). The cohort effect showed that prevalence risk gradually increased from 1897 to 1901 (RR = 0.91) to a peak in 1922–1926 (RR = 1.15) and then decreased (Figure 4B). The patterns for men and women were essentially identical, showing similar trends (Supplementary Tables S4, S5; Figure 4).

### Decomposition analysis

To evaluate the influence of population growth, aging, and epidemiological shifts on the burden of subarachnoid hemorrhage in middle-aged and elderly individuals, we conducted a decomposition analysis. The results demonstrated that population growth was the primary driver of the observed increase in cases over the past 32 years.

For incidence, the total increase was 166,339 cases, with population aging contributing 14,932 cases (8.98%) and population growth contributing 316,513 cases (190.28%). In contrast, epidemiological changes resulted in a decrease of 165,106 cases (−99.26%). For prevalence, the total increase was 2,579,779 cases, with population aging accounting for 54,484 cases (2.11%), population growth for 3,286,966 cases (127.41%), and epidemiological changes for a decrease of 761,670 cases (−29.53%).

**Figure 5:**
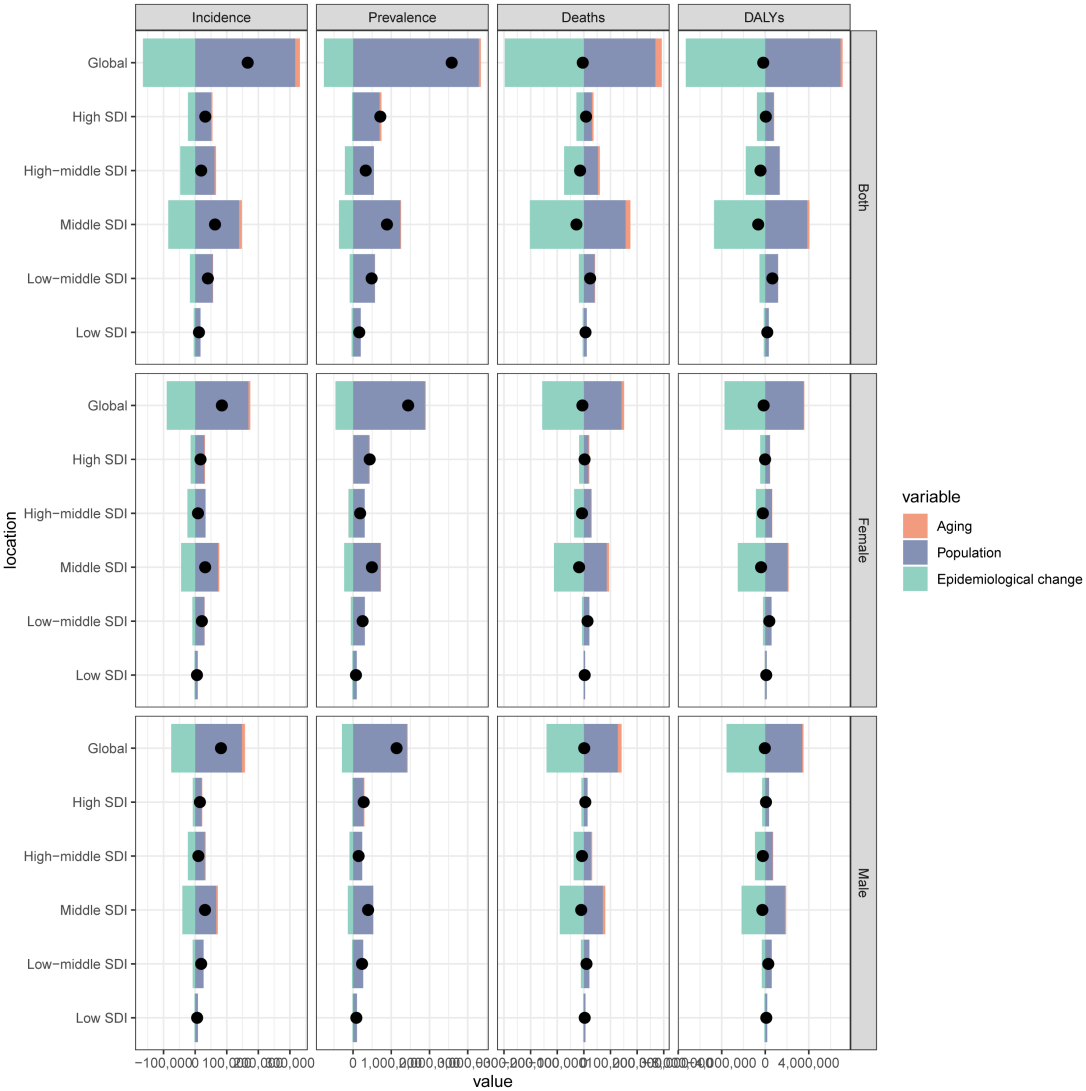
Decomposition analysis of changes in subarachnoid hemorrhage burden (incidence, prevalence, mortality, and disability-adjusted life years) among middle-aged and elderly populations from 1990 to 2021, attributable to population growth, population aging, and epidemiological changes across socio-demographic index (SDI) quintiles.

The total mortality decrease was 4,274 cases, with population aging accounting for a reduction of 23,887 cases (−558.87%), population growth for an increase of 268,506 cases (6,282.07%), and epidemiological changes for a decrease of 296,667 cases (−6,940.94%). The total DALYs decrease was 184,841 years, with population aging resulting in a decrease of 203,038 years (−109.85%), population growth leading to an increase of 6,911,373 years (3,739.08%), and epidemiological changes leading to a decrease of 7,299,253 years (−3,948.93%). In high SDI areas, the 2021 burden included 32,054 incident cases, 713,120 prevalent cases, 8,080 deaths, and 53,538 DALYs (Supplementary Tables S6–S9; Figure 5).

### Relationship between subarachnoid hemorrhage burden and socio-demographic development

The analysis demonstrated correlations between the SDI of various regions and countries in 2021 and the age-standardized rates of subarachnoid hemorrhage in adults. There was an inverted “U”-shaped relationship between ASMR and SDI. When SDI was less than 0.6, ASMR increased gradually, reached a peak when SDI was close to 0.6, and then began to decrease. Overall, there was a negative correlation between SDI and ASMR across the five SDI regions (*ρ* = −0.212, *p* = 0.002) (Supplementary Figure 4G). This trend was also reflected in the correlation between ASDR and SDI (Supplementary Figure 4H). However, among the five SDI regions, the correlations between SDI and ASIR and ASPR were weak (Supplementary Figures 4E,F).

**Figure 6:**
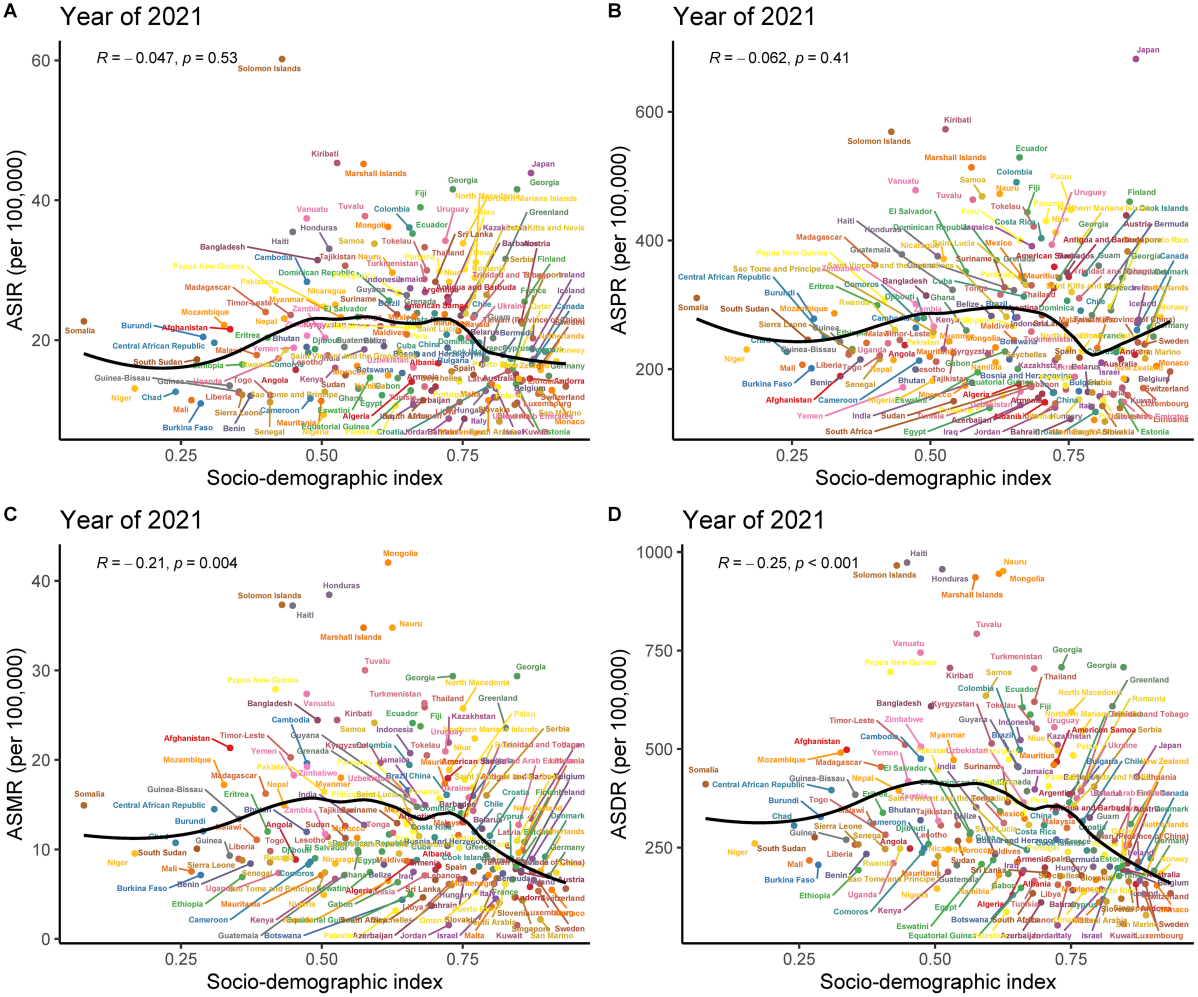
Pearson correlation analysis between socio-demographic index (SDI) and age-standardized rates of subarachnoid hemorrhage among middle-aged and elderly populations across 204 countries and territories in 2021. **(A)** Age-standardized incidence rate; **(B)** Age-standardized prevalence rate; **(C)** Age-standardized mortality rate; **(D)** Age-standardized DALYs rate.

A positive correlation was observed between SDI and ASPR among the 21 GBD regions (R = 0.095, *p* = 0.008). Conversely, negative correlations were observed between SDI and ASMR (R = −0.104, *p* = 0.003) and ASDR (R = −0.125, *p* < 0.001) (Supplementary Figures 5B–D), while a weak correlation was evident between SDI and ASIR (Supplementary Figure 5A). Further analysis of data from 204 countries and regions revealed that SDI was negatively correlated with ASMR (R = −0.21, *p* = 0.004) and ASDR (R = −0.25, *p* < 0.001) (Figures 6C,D), and exhibited weak correlations with ASIR and ASPR (Figures 6A,B).

### Health inequality analysis

**Figure 7:**
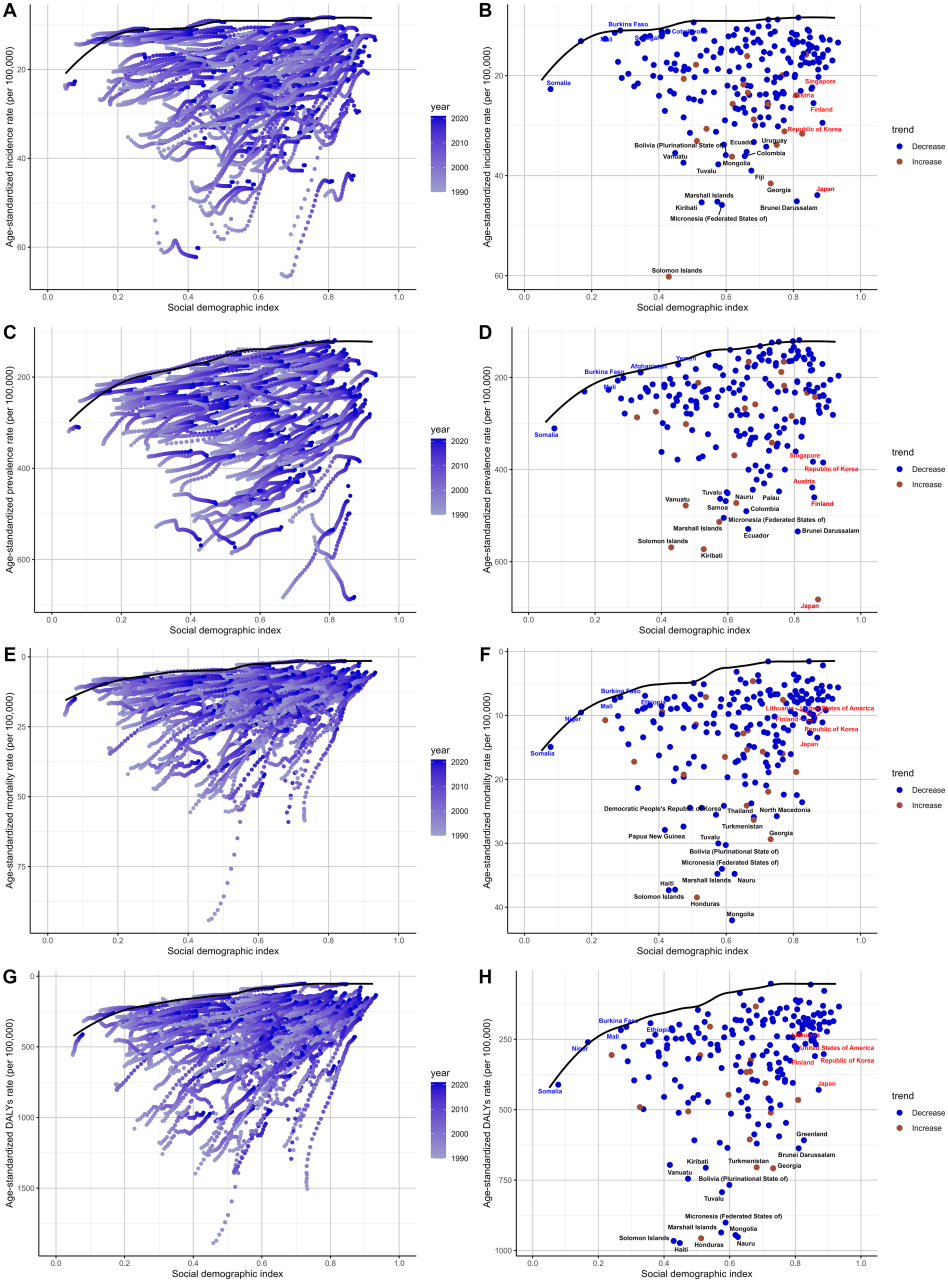
Health inequality analysis of subarachnoid hemorrhage burden among middle-aged and elderly populations in 1990 and 2021. **(A,B)** Slope Inequality Index (SII) of age-standardized incidence rate (ASIR) and age-standardized mortality rate (ASMR), respectively, showing absolute inequality trends over time. **(C,D)** Concentration Index (CI) of age-standardized prevalence rate (ASPR) and ASMR, respectively, reflecting relative inequality across socio-demographic index (SDI) levels. **(E,F)** SII and CI of age-standardized disability-adjusted life years (DALYs) rate (ASDR), respectively. **(G,H)** SII and CI of DALYs rate in 1990 and 2021, demonstrating a shift from mild inequality to pronounced inequality, especially in low-SDI regions. Positive SII values indicate greater burden in higher-SDI countries, while negative values indicate greater burden in lower-SDI countries. The CI ranges from -1 to 1, where values close to zero indicate equity, and more extreme values indicate disproportionate burden. These findings illustrate evolving global health disparities in subarachnoid hemorrhage burden.

The analysis of subarachnoid hemorrhage burden distribution among middle-aged and elderly people in 204 countries and territories revealed absolute and relative inequalities associated with the SDI. These inequalities were more pronounced in countries with higher SDI. The inequality slope index demonstrated that between 1990 and 2021, the discrepancy in prevalence per 100,000 individuals between countries with the highest and lowest SDI diminished from 40.474 (95% CI: −2.41 to 83.357) to 3.907 (95% CI: −34.892 to 42.705) (Figure 7C; Supplementary Table S10). Conversely, the concentration index declined from 0.049 (95% CI: 0.026 to 0.073) to 0.04 (95% CI: 0.005 to 0.075) over the same period (Figure 7D; Supplementary Table S10).

For DALYs, the inequality slope index declined from 1.694 (95% CI: −87.228 to 90.617) in 1990 to −88.866 (95% CI: −151.094 to −26.638) in 2021. Concurrently, the concentration index increased from −0.017 (95% CI: −0.072 to 0.039) to −0.018 (95% CI: −0.046 to 0.01) (Figures 7G,H; Supplementary Table S10). No significant temporal changes were observed for ASIR and ASMR (Figures 7A,B,E,F).

### Projections of subarachnoid hemorrhage burden to 2040

**Figure 8:**
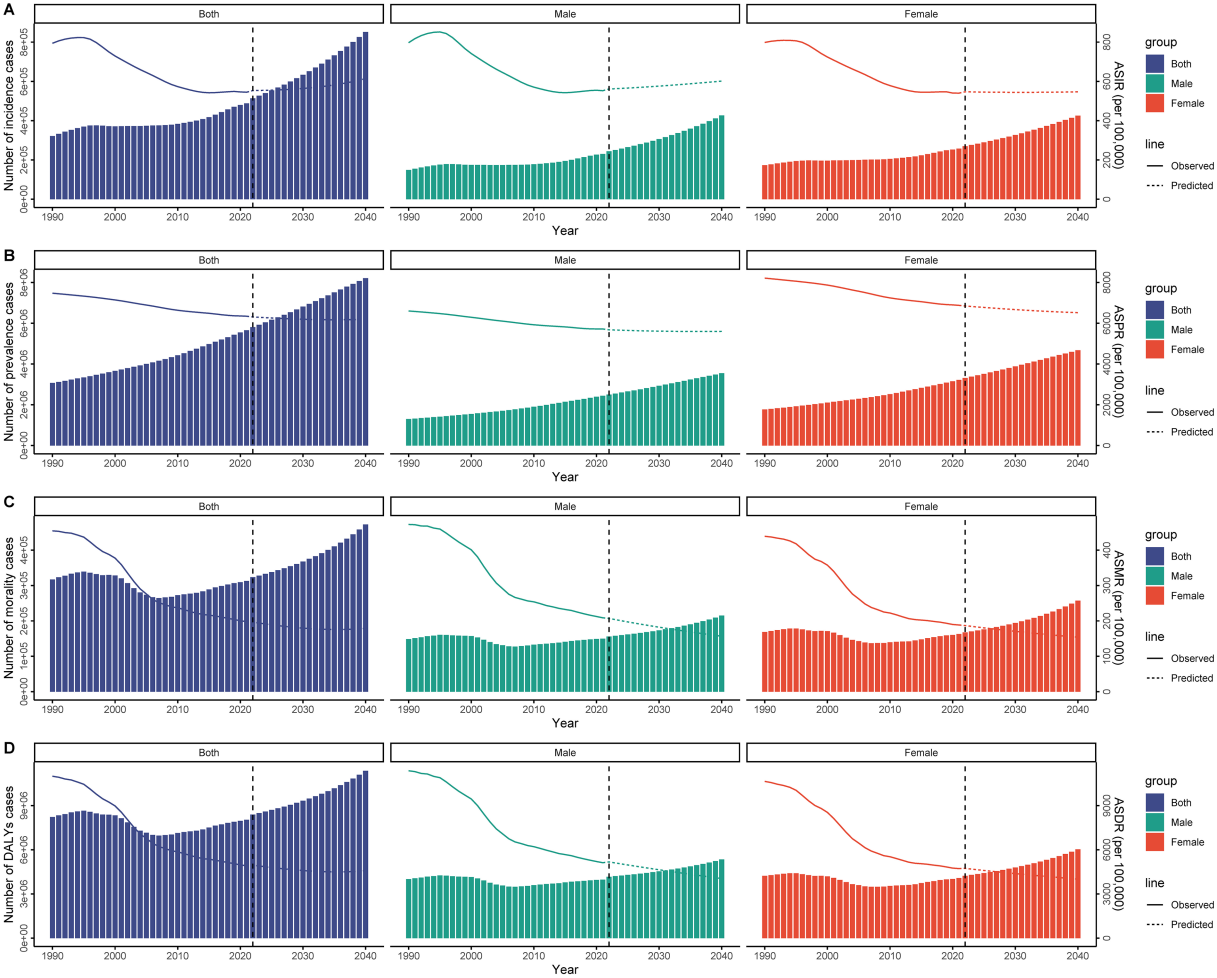
Projected global burden of subarachnoid hemorrhage among middle-aged and elderly populations from 1990 to 2040, showing case numbers and age-standardized rates. **(A)** Incidence cases and age-standardized incidence rate; **(B)** Prevalence cases and age-standardized prevalence rate; **(C)** Deaths and age-standardized mortality rate; **(D)** Disability-adjusted life years and age-standardized DALYs rate.

According to the analysis and projection from 2022 to 2040, the burden of subarachnoid hemorrhage in the elderly shows divergent trends across different measures. The results indicate that the absolute numbers of cases will increase significantly for incidence, prevalence, mortality, and DALYs. A slight increase in ASIR is expected. In contrast, the ASPR, ASMR, and ASDR show significant downward trends. The same patterns are observed for both men and women (Figure 8).

### Frontier analysis

**Figure 9:**
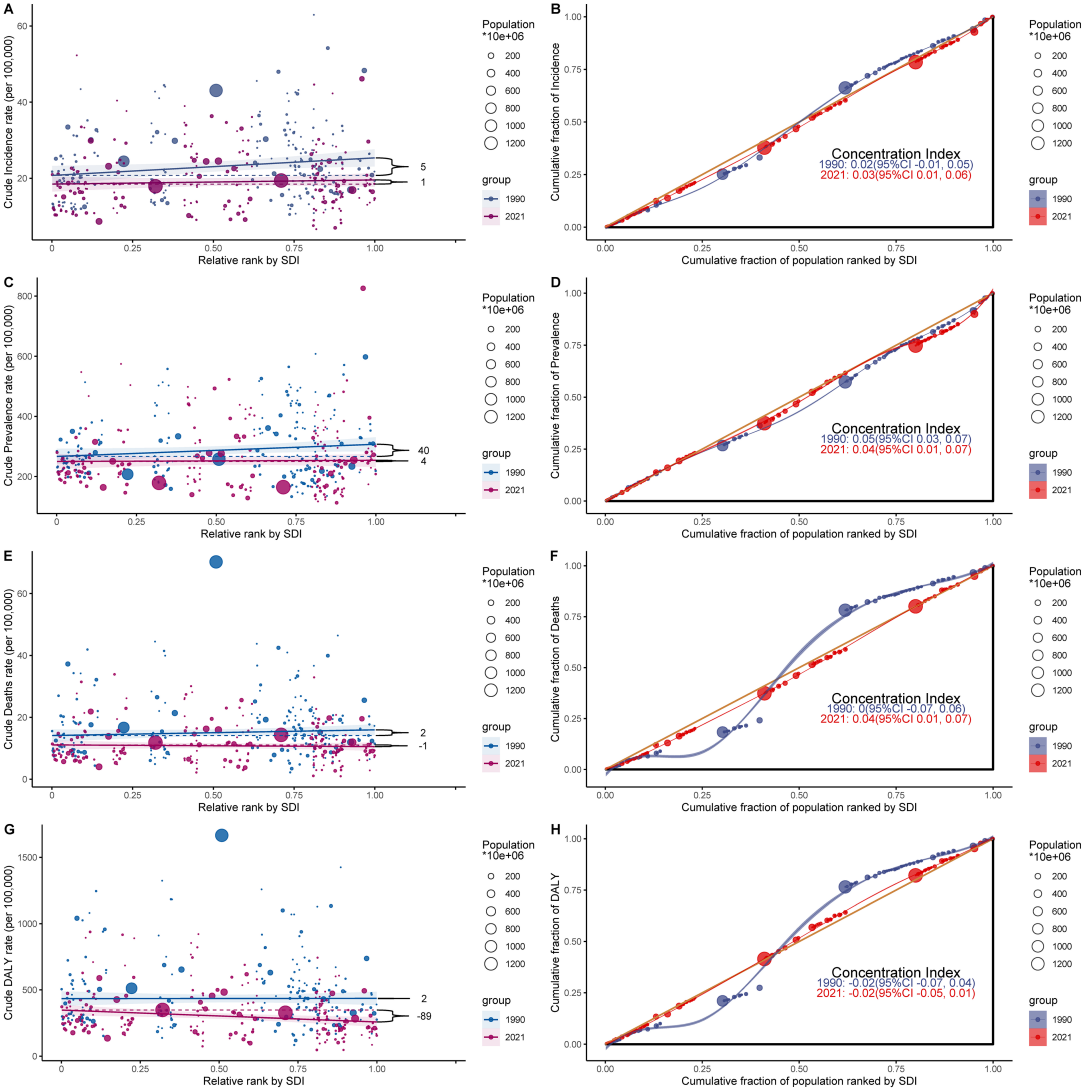
Frontier analysis of the relationship between socio-demographic index (SDI) and subarachnoid hemorrhage burden among middle-aged and elderly populations in 2021. **(A,B)** Frontier analysis of age-standardized incidence rate; **(C,D)** Frontier analysis of age-standardized prevalence rate; **(E,F)** Frontier analysis of age-standardized mortality rate; **(G,H)** Frontier analysis of age-standardized DALYs rate.

Frontier analysis of the SDI and subarachnoid hemorrhage burden among adults from 204 countries and regions between 1990 and 2021 reveals differences in trends across regions. The results show that disease burden demonstrates a significant downward trend over time, with the frontier gradually shifting toward lower burden levels (Figure 9A). Similarly, prevalence rates gradually decreased as SDI levels increased (Figure 9C). Mortality rates and DALYs showed similar trends, further indicating that the global burden of subarachnoid hemorrhage in middle-aged and elderly people gradually decreased between 1990 and 2021 (Figures 9E,G).

The 2021 frontier analysis highlights significant differences between countries and regions. There are substantial differences in incidence rates even at similar SDI levels. The ASIR shows a decreasing trend as SDI values increase and stabilizes after SDI values exceed 0.8. Some countries show significant differences in the effectiveness of control strategies for middle-aged and older people with subarachnoid hemorrhage. For example, Somalia, Burkina Faso, and Saudi Arabia had relatively good control effects in 2021, with effectiveness differences of 0, 0.078, and 0.112, respectively. In contrast, Solomon Islands, Federated States of Micronesia, and Brunei Darussalam had poor control effects, with efficacy differences of 50.218, 36.823, and 36.676, respectively, significantly different from the frontier (Figure 9B; Supplementary Table S11). Similar results are shown in the analysis of ASPR, ASMR, and ASDR (Figures 9D,F,H; Supplementary Tables S12–S14).

### Association between health workforce and subarachnoid hemorrhage burden

**Figure 10:**
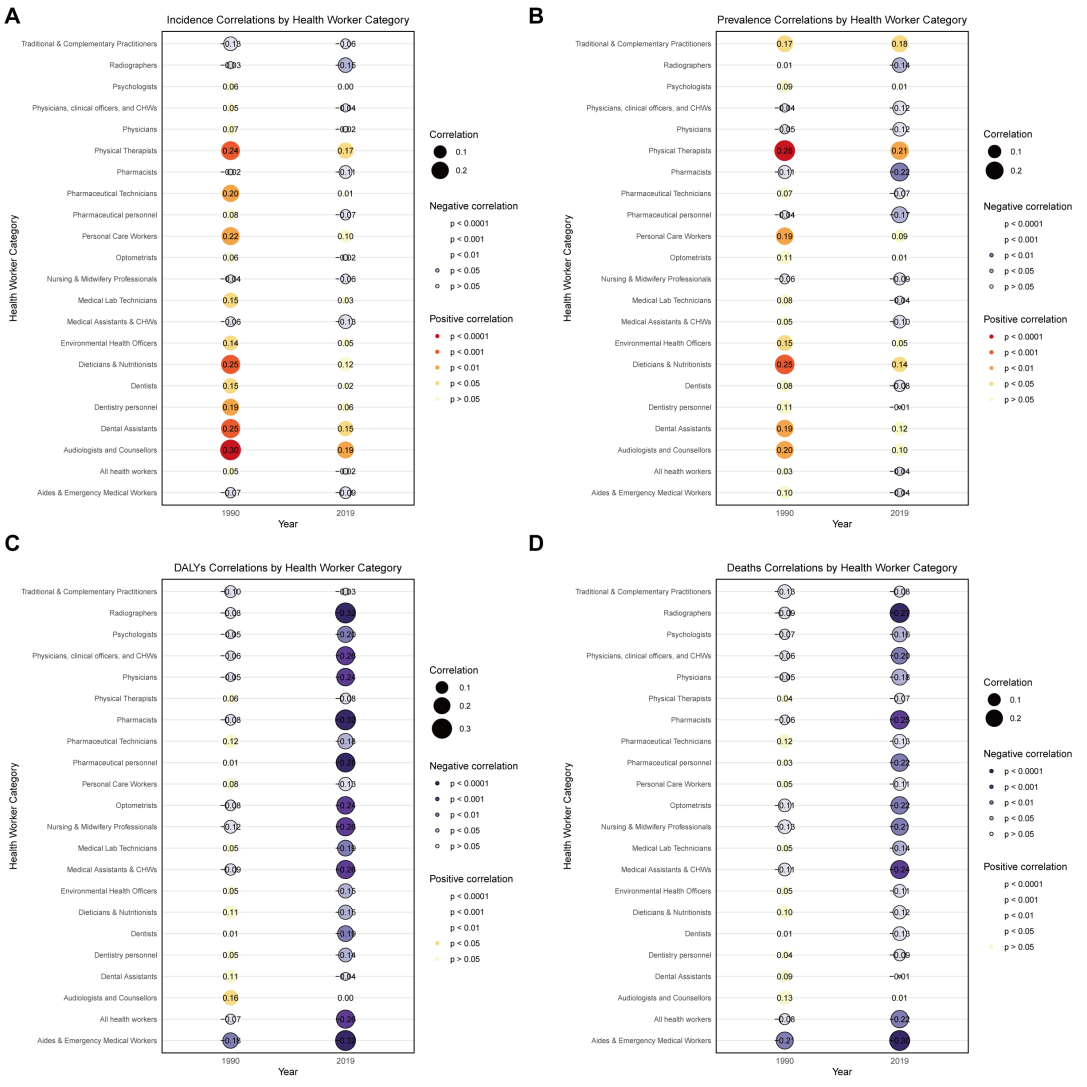
Global correlations between health worker categories and age-standardized disease burden measures of subarachnoid hemorrhage among adults aged ≥ 45 years across 204 countries and territories in 1990 and 2019. **(A)** Incidence rates; **(B)** Prevalence rates; **(C)** Disability-adjusted life years (DALYs) rates; **(D)** Death rates. Circle size indicates correlation magnitude. Filled circles represent positive correlations (warm colors); bordered circles represent negative correlations (cool colors). Numbers represent Spearman correlation coefficients. Color intensity indicates statistical significance levels (*p* < 0.0001 to *p* > 0.05).

To further elucidate the relationship between health workforce distribution and subarachnoid hemorrhage burden, we utilized correlation analysis to examine associations between 22 different health worker categories and four disease burden measures across 204 countries and territories in 1990 and 2019. The correlation analysis revealed distinct patterns that evolved substantially over the 30-year period (Figures 10A–D).

In 1990, correlations were generally weak and inconsistent across different health worker categories, with most correlations ranging from −0.21 to 0.30. However, by 2019, more pronounced and statistically significant associations emerged, particularly for mortality-related outcomes. Emergency medical workers and aides demonstrated the strongest protective associations, with correlations strengthening dramatically from r = −0.21 (*p* = 0.0028) for deaths in 1990 to r = −0.30 (*p* < 0.001) in 2019, and DALYs correlations intensifying from r = −0.18 (*p* = 0.0088) to r = −0.32 (*p* < 0.001).

Conversely, rehabilitation-oriented health workers such as physical therapists showed consistent positive correlations with prevalence (r = 0.28, *p* < 0.001 in 1990; r = 0.21, *p* = 0.0031 in 2019) and incidence measures (r = 0.24, *p* < 0.001 in 1990; r = 0.17, *p* = 0.016 in 2019). Analysis of extreme cases revealed striking disparities: countries with the highest subarachnoid hemorrhage death rates, such as China (94.2 per 100,000 in 1990) and Mongolia (45.4 per 100,000 in 2019), had remarkably low emergency medical worker densities of 0.47 and 11.2 per 10,000 population, respectively. In contrast, countries with the lowest death rates, including Jordan (2.9 per 100,000 in 1990; 1.5 per 100,000 in 2019) and Kuwait (1.5 per 100,000 in 2019), maintained substantially higher emergency workforce levels of 1.7–26.1 per 10,000 population. These findings suggest that negative correlations indicate protective workforce capacity that can effectively suppress disease burden, warranting increased investment, while positive correlations may reflect demand-driven resource allocation rather than therapeutic efficacy, requiring careful evaluation of cost-effectiveness before expanding such services.

## Discussion

This study, based on data from 1990 to 2021, highlights differences in burden across countries and regions and the drivers behind them. Despite differences in SAH burden between countries, the overall burden among middle-aged and older adults has increased significantly globally since 1990, especially in countries with a medium Social Development Index. Disaggregation analyses show that population growth and aging are the main factors driving the increasing burden of SAH, while health inequality analyses show that countries with a high SDI have a higher burden, but this inequality has decreased over time. Although the ASR of SAH prevalence, mortality and DALYs is expected to decrease by 2040, the absolute number of cases will continue to increase due to global population aging, indicating that there will be significant challenges in controlling and managing SAH in middle-aged and elderly people.

The burden of SAH in middle-aged and elderly people varies significantly among SDI regions. The burden is highest in the middle SDI region due to rapid urbanization, an aging population, and the widespread presence of risk factors such as hypertension. For example, China, a typical medium SDI country, has experienced a sharp increase in air pollution (especially PM2.5) along with rapid urbanization and industrialization, which has become a major driver of the country’s health burden (26–28). Studies have shown that PM2.5 significantly increases the risk of cardiovascular and cerebrovascular disease by inducing chronic inflammation and vascular dysfunction, especially in the middle-aged and elderly population, which has contributed to the continued increase in the burden of SAH in China (29, 30). In addition, the Westernized diet and increased intake of high-fat and high-salt foods have further increased the prevalence of hypertension and diabetes among middle-aged and elderly people in China, exacerbating the burden of SAH (31). Nevertheless, these countries have significantly reduced SAH mortality and DALYs by strengthening healthcare systems, effectively managing hypertension, and promoting advanced treatment techniques such as endovascular embolization. This suggests that despite the high burden of SAH in middle-income countries, effective medical interventions play a key role in reducing the burden.

In contrast, middle-aged and elderly populations in low-to-medium SDI regions have a lower incidence of SAH, but the burden of mortality and DALYs in these regions continues to rise due to lack of medical resources and delayed treatment. This is particularly evident in Mongolia, Haiti and the Pacific Island countries, where the healthcare infrastructure is very weak, especially in rural and remote areas. The lack of neuroimaging equipment and treatment techniques makes early screening and intervention difficult (32, 33). In addition, geographic isolation limits the introduction of outside medical assistance and advanced technology, making it difficult for patients to receive effective treatment in a timely manner (34), further increasing mortality and disability rates. These problems are compounded by inadequate health education. Many middle-aged and elderly patients are unable to recognize the early symptoms of SAH in time, which delays medical treatment and worsens the disease burden. In contrast, although the incidence and prevalence of SAH are high in high-SDI and high-to-moderate SDI countries, the rapid development of precision medicine has enabled these countries to identify high-risk patients earlier and more accurately through innovative medical intervention strategies (6); in particular, countries such as Japan have further developed robotic surgery-based techniques for minimally invasive endovascular embolization, which significantly reduce surgical risks and recurrence rates (35).

In an analysis of age and gender differences in the burden of subarachnoid hemorrhage (SAH) in middle-aged and elderly people, it was found that the age group with the highest burden of subarachnoid hemorrhage was 50 to 69 years. Which is closely related to the long-term accumulation of chronic diseases such as atherosclerosis, increased vascular fragility, and hypertension and diabetes, which are common at this stage (36). Decreased elasticity of the arterial wall leads to an increased risk of aneurysm formation and SAH due to the increased susceptibility of blood vessels to rupture (37). The study also showed that women aged 65 years and older had a significantly higher burden of SAH than men, especially in terms of mortality and DALYs. This difference may be related to the decrease in estrogen levels after menopause, which provides women with vasoprotective effects and reduces the fragility of the vascular wall prior to menopause. After menopause, the ability of women’s blood vessels to recover is weakened, increasing the risk of aneurysm formation and rupture (38). In addition, lifestyle changes in older women (such as reduced physical activity and irregular diet) may also increase the risk of SAH (39). Gender differences and pathological mechanisms specific to middle-aged and elderly people have a significant impact on the treatment effect and prognosis in the treatment and rehabilitation of SAH (40, 41). Minimally invasive endovascular embolization has become the preferred treatment for aneurysmal SAH, especially in elderly patients with impaired physical fitness, because it is less invasive. However, postmenopausal women have a weaker vascular repair capacity, a slower recovery, and a higher incidence of postoperative complications (42). They are more sensitive to antihypertensive and anticoagulant medications and require more frequent adjustment of treatment plans (43). In contrast, male patients have more stable postoperative drug tolerance, but inadequate lifestyle adjustments lead to a higher risk of recurrence (11). In addition, women have slower recovery of neurological function and more frequent psychological problems such as depression and anxiety, further delaying the recovery process and highlighting the importance of gender-specific psychological interventions (44).

Our novel analysis of health workforce categories revealed significant associations between specific health worker types and SAH burden that evolved substantially over the 30-year study period. The strengthening inverse relationship between emergency medical workers and SAH mortality outcomes (correlation improving from r = −0.21 in 1990 to r = −0.30 in 2019) provides compelling evidence that emergency care capacity is a critical determinant of SAH outcomes. Countries with the lowest SAH mortality rates, such as Jordan and Kuwait, maintained substantially higher emergency medical workforce densities (1.7–26.1 per 10,000 population) compared to high-mortality countries like China and Mongolia (0.47–11.2 per 10,000 population). This relationship likely reflects the time-sensitive nature of SAH management, where rapid access to emergency care, advanced imaging, and neurosurgical intervention can dramatically improve survival outcomes. The observed strengthening of this association over time suggests that as emergency medical systems have become more sophisticated and standardized globally, the protective effect of adequate emergency workforce capacity has become more pronounced. Conversely, the positive correlations observed between rehabilitation workers and SAH prevalence measures reflect demand-driven resource allocation rather than preventive effectiveness. Countries with higher SAH prevalence naturally require more rehabilitation services to manage survivors’ long-term neurological deficits, explaining why physical therapist density correlates positively with disease burden. This finding has important policy implications: while emergency medical workforce expansion represents a direct intervention to reduce SAH mortality, rehabilitation workforce planning should be viewed as a necessary response to existing disease burden rather than a primary prevention strategy.

When analyzing the effect of period, the study found that with the advancement of medical technology and the strengthening of risk management, the incidence and mortality rates of SAH in middle-aged and elderly people have gradually decreased. The widespread use of imaging techniques (such as CT angiography) and minimally invasive surgery has significantly improved the rate of early diagnosis, reducing acute mortality and the risk of long-term disability. At the same time, public health awareness and better management of risk factors such as hypertension have also reduced the incidence and recurrence of SAH (45). These improvements in prevention and treatment worldwide have played an important role in reducing the burden of SAH. Studies of birth cohort effects have shown that earlier birth cohorts have a higher risk of SAH, which may be related to poorer medical conditions and living environments, while the risk of SAH in later birth cohorts has gradually decreased due to better public health and medical conditions (46).

Our frontier analysis quantified the relationship between socio-demographic development and SAH burden, revealing significant variations in control effectiveness across countries at similar development levels. The frontier represents the optimal performance achievable at each SDI level, with distances from the frontier indicating relative efficiency in disease control. Somalia and Burkina Faso demonstrated exemplary control effectiveness (efficiency differences of 0 and 0.078, respectively) despite their low SDI status, suggesting that effective SAH management strategies can be implemented even in resource-constrained settings. Several factors may explain the superior performance of these low SDI countries. First, Somalia’s protracted conflict may have paradoxically led to the development of efficient trauma and emergency care systems focused on life-threatening conditions, creating infrastructure that benefits SAH management. Second, both countries may benefit from targeted international health assistance programs that specifically address emergency neurological care. Third, their relatively young population structures may result in SAH cases occurring primarily in younger, more resilient patients with better prognosis. Additionally, traditional dietary patterns in these regions may provide some cardiovascular protection compared to the Westernized diets increasingly common in middle SDI countries. In contrast, countries like Solomon Islands, Micronesia, and Brunei showed poor control effectiveness (efficiency differences of 50.218, 36.823, and 36.676, respectively) despite varying SDI levels. These countries face unique challenges including geographic isolation, limited medical infrastructure, and difficulties in establishing comprehensive emergency care networks across dispersed populations. For Solomon Islands and Micronesia, the small population sizes and extreme geographic fragmentation make it economically challenging to maintain specialized neurosurgical capabilities on every inhabited island. The success of high SDI countries like Saudi Arabia and South Korea in leveraging telemedicine and artificial intelligence represents one pathway for improvement, but may not be directly applicable to low SDI settings due to infrastructure and cost constraints. Instead, low SDI countries might benefit more from: (1) regional medical cooperation networks that allow for rapid patient transfer and specialist consultation; (2) focused training programs for general practitioners in SAH recognition and initial management; (3) simplified treatment protocols adapted to resource constraints; and (4) mobile health units that can provide emergency care in remote areas. The examples of Somalia and Burkina Faso suggest that with appropriate strategies tailored to local contexts, even low-resource settings can achieve effective SAH control.

Despite the comprehensive data analysis provided by this study, there are several limitations. First, poor data quality in low-income countries, especially in remote areas, is due to the lack of complete surveillance systems and case registration, which may bias estimates of disease burden and affect extrapolation of results. In the future, data collection capacity in these areas should be improved through increased global collaboration, the promotion of standardized surveillance tools, and the use of mobile data collection methods to ensure the representativeness and reliability of research results (47). In addition, global health organizations (e.g., the World Health Organization and the United Nations) should increase technology transfer and training in low-resource settings to promote standardized surveillance systems (48). Second, despite the use of advanced statistical methods such as Bayesian regression and age-period-cohort models, predictive analytics still faces challenges. Uncertainties such as demographic changes, global economic fluctuations, and public health emergencies (e.g., the COVID-19 pandemic) may have unpredictable effects on future disease burdens. To account for these complexities, future research should consider more dynamic models and regularly update data to improve the accuracy of projections. Meanwhile, scenario analysis and sensitivity analysis should be further developed to help identify key factors and potential pathways of change that affect prediction outcomes.

## Conclusion

This study demonstrates dynamic trends in the global burden of subarachnoid hemorrhage among middle-aged and older people between 1990 and 2021. Although age-standardized rates are decreasing worldwide, absolute case numbers continue to increase with population growth and aging, particularly in low- and middle-income countries where mortality and disability-adjusted life years burden remains significant. Our novel health workforce analysis provides the first global evidence that emergency medical workforce capacity represents a modifiable determinant of SAH outcomes, with countries maintaining higher emergency care worker densities achieving significantly lower mortality rates. Frontier analysis revealed that effective SAH control can be achieved across different socio-demographic development levels, with countries like Somalia and Burkina Faso demonstrating successful management strategies despite resource constraints, while others perform below their potential. Projections to 2040 suggest continued increases in absolute case numbers despite declining age-standardized rates, emphasizing the need for enhanced emergency medical workforce capacity and regional cooperation networks to manage the growing burden and reduce global SAH disparities through evidence-based healthcare resource allocation.

## Data Availability

The original contributions presented in the study are included in the article/Supplementary material, further inquiries can be directed to the corresponding author.

## Supplementary material

SUPPLEMENTARY FIGURE 1 Temporal trends of age-standardized incidence, prevalence, mortality, and DALYs rates of subarachnoid hemorrhage stratified by sex across 21 GBD regions from 1990 to 2021.

SUPPLEMENTARY FIGURE 2 Regional variations in temporal trends of age-standardized incidence, prevalence, mortality, and DALYs rates of subarachnoid hemorrhage by sex across 21 GBD regions from 1990 to 2021.

SUPPLEMENTARY FIGURE 3 Global distribution maps of age-standardized incidence, prevalence, mortality, and disability-adjusted life years (DALYs) rates of subarachnoid hemorrhage among middle-aged and elderly populations in 1990 and 2021. **(A,E)** Incidence rates; **(B,F)** Prevalence rates; **(C,G)** Mortality rates; **(D,H)** Disability-adjusted life years rates.

SUPPLEMENTARY FIGURE 4 Pearson correlation analysis between socio-demographic index (SDI) quintiles and age-standardized rates of subarachnoid hemorrhage among middle-aged and elderly populations in 1990 and 2021. **(A,E)** Age-standardized incidence rate; **(B,F)** Age-standardized prevalence rate; **(C,G)** Age-standardized mortality rate; **(D,H)** Age-standardized DALYs rate.

SUPPLEMENTARY FIGURE 5 Pearson correlation analysis between socio-demographic index (SDI) and age-standardized rates of subarachnoid hemorrhage among middle-aged and elderly populations at the Global Burden of Disease regional level from 1990 to 2021. **(A)** Age-standardized incidence rate; **(B)** Age-standardized prevalence rate; **(C)** Age-standardized mortality rate; **(D)** Age-standardized DALYs rate.

SUPPLEMENTARY TABLE S1 Age-specific incidence, prevalence, mortality, and disability-adjusted life years (DALYs) associated with middle-aged and old-aged subarachnoid hemorrhage and the corresponding age-standardized rates for 204 countries between 1990 and 2021. EAPC estimated percentage change annually; UI uncertainty interval.

SUPPLEMENTARY TABLE S2 Middle-aged and elderly-related morbidity, prevalence, mortality, and disability-adjusted life years associated with subarachnoid hemorrhage by age from 1990 to 2021 and the corresponding age-standardized rates. UI, uncertainty interval.

SUPPLEMENTARY TABLE S3 An age-period-group analysis of the risk of subarachnoid hemorrhage in middle-aged and elderly in both sexes.

SUPPLEMENTARY TABLE S4 Age-period-group analysis of the relative risk of subarachnoid hemorrhage in middle-aged and elderly in males.

SUPPLEMENTARY TABLE S5 Age-period-cohort analysis of the relative risk of subarachnoid hemorrhage in middle-aged and elderly in females.

SUPPLEMENTARY TABLE S6 Changes in the incidence of middle-aged and elderly subarachnoid hemorrhage between 1990 and 2021, based on population factors.

SUPPLEMENTARY TABLE S7 Changes in the prevalence of middle-aged and elderly subarachnoid hemorrhage between 1990 and 2021, based on population factors.

SUPPLEMENTARY TABLE S8 Changes in the mortality of middle-aged and elderly subarachnoid hemorrhage between 1990 and 2021, based on population factors.

SUPPLEMENTARY TABLE S9 Changes in the DALYs of middle-aged and elderly subarachnoid hemorrhage between 1990 and 2021, based on population factors.

SUPPLEMENTARY TABLE S10 Absolute and relative inequality index of incidence, prevalence, mortality, and DALYs in all regions for both sexes, 1990 vs 2021.

SUPPLEMENTARY TABLE S11 Frontier analysis and effective differences in the incidence of middle-aged and elderly subarachnoid hemorrhage by country or region.

SUPPLEMENTARY TABLE S12 Frontier analysis and effective differences in the prevalence of middle-aged and elderly subarachnoid hemorrhage by country or region.

SUPPLEMENTARY TABLE S13 Frontier analysis and effective differences in the mortality of middle-aged and elderly subarachnoid hemorrhage by country or region.

SUPPLEMENTARY TABLE S14 Frontier analysis and effective differences in the DALYs of middle-aged and elderly subarachnoid hemorrhage by country or region.

SUPPLEMENTARY TABLE S15 Global incidence, prevalence, mortality, and disability-adjusted life years for subarachnoid hemorrhage in the elderly, and the corresponding age-standardized rates, from 1990 to 2021. EAPC estimated percentage change annually; UI uncertainty interval.
