# Supplementary figures and images for: Insights into the epidemiological analysis of subarachnoid hemorrhage burden and trends in middle-aged and elderly populations: a global perspective from the Global Burden of Disease Study 2021
Source: Front Neurol. 2025 Aug 18;16:1518319. doi: 10.3389/fneur.2025.1518319 (PMC12400870; doi:10.3389/fneur.2025.1518319)

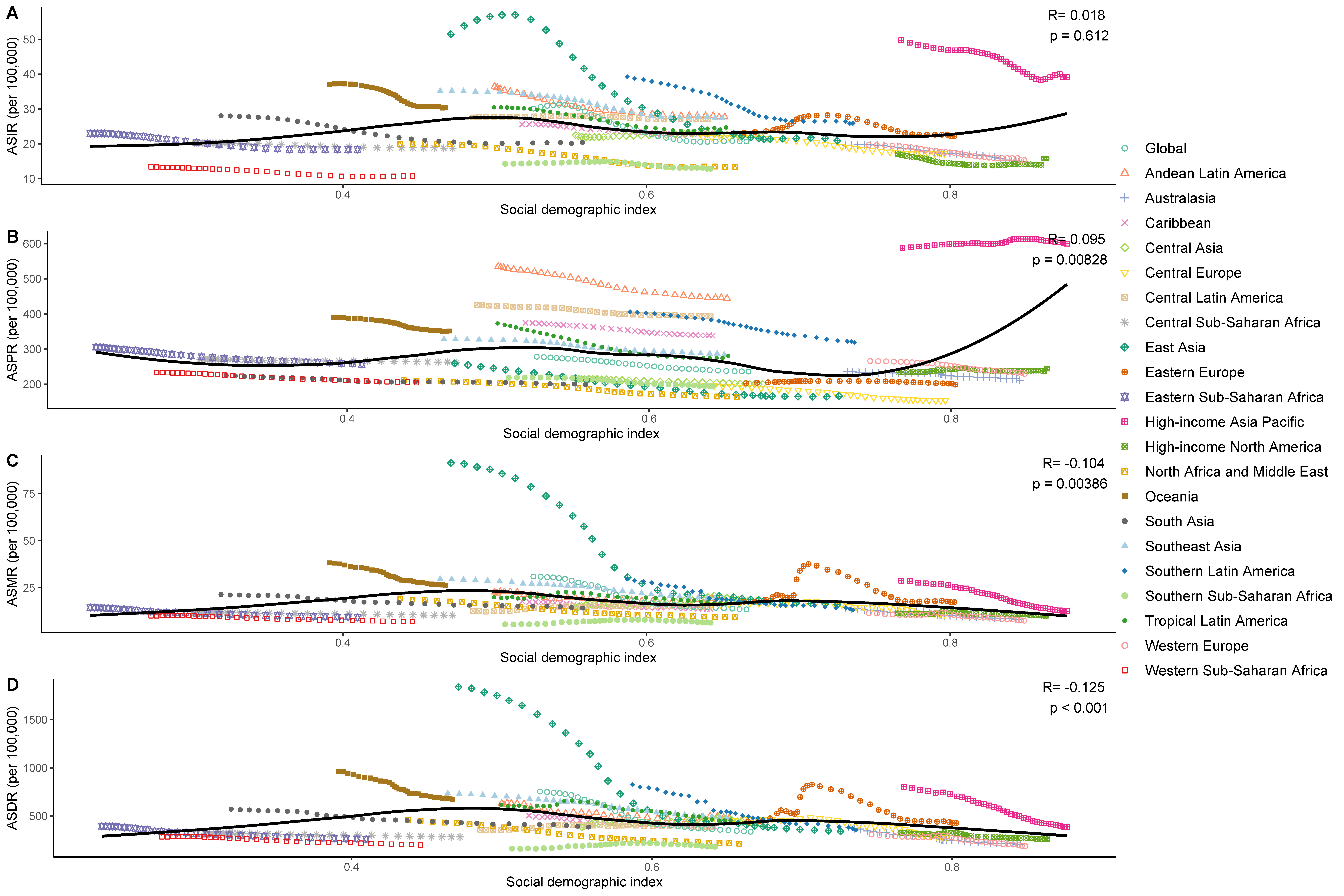

Supplement: SUPPLEMENTARY FIGURE 5 — Pearson correlation analysis between socio-demographic index (SDI) and age-standardized rates of subarachnoid hemorrhage among middle-aged and elderly populations at the Global Burden of Disease regional level from 1990 to 2021. (A) Age-standardized incidence rate; (B) Age-standardized prevalence rate; (C) Age-standardized mortality rate; (D) Age-standardized DALYs rate. [file Image_5.TIFF]
